# Caucasian and African American racial disparity in neonatal hospital mortality

**DOI:** 10.3389/fped.2024.1289399

**Published:** 2024-03-04

**Authors:** Maria Burdjalov, Ibrahim Qattea, Vanishree Nandakumar, Mohamed A. Mohamed, Hany Aly

**Affiliations:** ^1^ College of Arts and Sciences, The Ohio State University, Columbus, OH, United States; ^2^ Neonatology, Cleveland Clinic Children’s Hospital, Cleveland, OH, United States

**Keywords:** disparity, infant, mortality, neonatal mortality, race

## Abstract

**Objective:**

To examine disparity in hospital mortality among Caucasian (C) and African American (AA) neonates born at different gestational ages (GA).

**Methods:**

De-identified national inpatient data were obtained from the Healthcare Cost and Utilization Project (HCUP) from the Agency for Healthcare Research and Quality (AHRQ) for the years 2011–2018. We compared the odds ratio for mortality among C and AA infants by sex and GA category. Analyses were repeated after controlling for multiple maternal and neonatal confounding variables in a logistic regression model.

**Results:**

The study included 18,758,233 infants; 78.3% of them were C and 21.7% were AA. Compared to C population, AA population has a significantly higher mortality in term infants born at GA ≥ 36 weeks. The racial/ethnic disparity in preterm infants was inconsistent without any difference at 35–36 weeks in male and female infants. The overall aOR for mortality in AA in all male preterm infants ≤36 weeks was 1.44 (1.39–1.49), <0.01; and the overall aOR for mortality in AA in all preterm female infants ≤36 weeks was 1.38 (1.33–1.44).

**Conclusion:**

Racial/Ethnic disparity in hospital mortality exists with higher AA mortality in infants born with GA > 36 weeks and less AA mortality in infants born with GA 24–26 weeks.

## 1 Introduction

Infant mortality is a measure of public health for a country and is an important measure in identifying risks, trends, and concerns within the healthcare sphere (1). In 2018, the United States infant mortality rate was 5.67 deaths per 1,000 live births. The inequitable distribution of socioeconomic conditions across populations contributes to pervasive health disparities (2). Research has shown that African American (AA) women have worse birth outcomes, regardless of socioeconomic position, compared to their Caucasian (C) counterparts. The persistence of racial/ethnic differences in infant's birth weight between mothers of low risk indicates a need for research that focuses beyond the traditional risk factors and towards economic, social, environmental, and medical conditions between AA and C populations (3).

There is an unmet need to investigate whether an ethnic disparity in neonatal mortality exists among AA and C population and how early in gestation it starts. We hypothesized that increased ethnic disparities within neonatal mortality are rooted early on in life at an earlier gestational age. In this study, we utilized a neonatal cohort from a national database to test the hypothesis that AA and C racial/ethnic disparities exist in neonatal population.

## 2 Patients and methods

This is an epidemiological study that used de-identified patient data obtained from the Healthcare Cost and Utilization Project (HCUP) from the Agency for Healthcare Research and Quality (AHRQ) for the years 2011–2018. This is the largest publicly available all-payer pediatric inpatient care database in the United States (4). HCUP develops the National Inpatient Sample (NIS) dataset every year, which includes 20% of the HCUP samples (5). The NIS is the largest publicly available inpatient care database in the United States. The Kids' Inpatient Database (KID) is the pediatric version of NIS and has been produced every three years (6). The NIS dataset was used for the years 2011–2018. The KID dataset was used for the years 2012, 2015 and 2018. Since the KID dataset is specific to pediatric and neonatal cases, it was used preferentially for data in the year it was released.

The study included all in-born or transferred-in neonates with assigned race of C or AA. According to HCUP coding, ethnic/racial determination of newborn is solely based on maternal race/ethnicity. The study hypothesis addressed C and AA only, therefore neonates who were Hispanic, Asian, American Indian, or Alaska Native, Native Hawaiian or Other Pacific Islander were excluded from the dataset. The primary outcome was in-hospital mortality for neonates admitted within the first 28 days of life. Neonates with anomalies known to associate with high mortality rate regardless of ethnicity were not include in the study. Therefore, the study excluded neonates with neural tube defects or other major brain anomalies, congenital diaphragmatic hernia, abdominal wall defects (gastroschisis or omphalocele), or other major congenital anomalies. In addition, infants with chromosomal disorders or major genetic diseases were excluded. To avoid duplication of records, neonates who were transferred were counted only once at the receiving hospital and were excluded from the sending hospital.

We compared the odds ratio for mortality among AA and C infants by sex and GA category. Data were represented as numbers and percentages for categorical variables and in means and medians for continuous parametric and non-parametric data, respectively. Categorical data were analyzed using Chi-square test and continuous variables were analyzed using *t*-test. Regression models were built to adjust for confounding variables. Patient demographics collected included ethnic group, sex, gestational age, birthweight, outcome of survival or death, length of stay. A total of 3 maternal factors (hypertension, diabetes, and chorioamnionitis) and 12 neonatal factors (sex, birthweight category, occurrence of hypoxic ischemic insults, intraventricular hemorrhage, periventricular leukomalacia, respiratory distress syndrome, persistent pulmonary hypertension, pulmonary hemorrhage, bronchopulmonary dysplasia, sepsis, necrotizing enterocolitis, and primary type of insurance) were used in the regression analyses. SAS 9.4 (Cary, NC) program was used for statistical analysis. *P*-values equal to or less than 0.05 were considered statistically significant.

This study was exempt from IRB approval**.**

Mortality in periviable infants with GA < 24 weeks is attributed to two different factors; one is a biological factor that determines the viability of the infant, and the other would be a socio/environmental factor on how aggressive the managing team and/or parents is willing to resuscitate their infant. In order to stratify these two factors, analyses of periviable infants were repeated after excluding mortalities within the first 48 h.

## 3 Results

**Figure 1:**
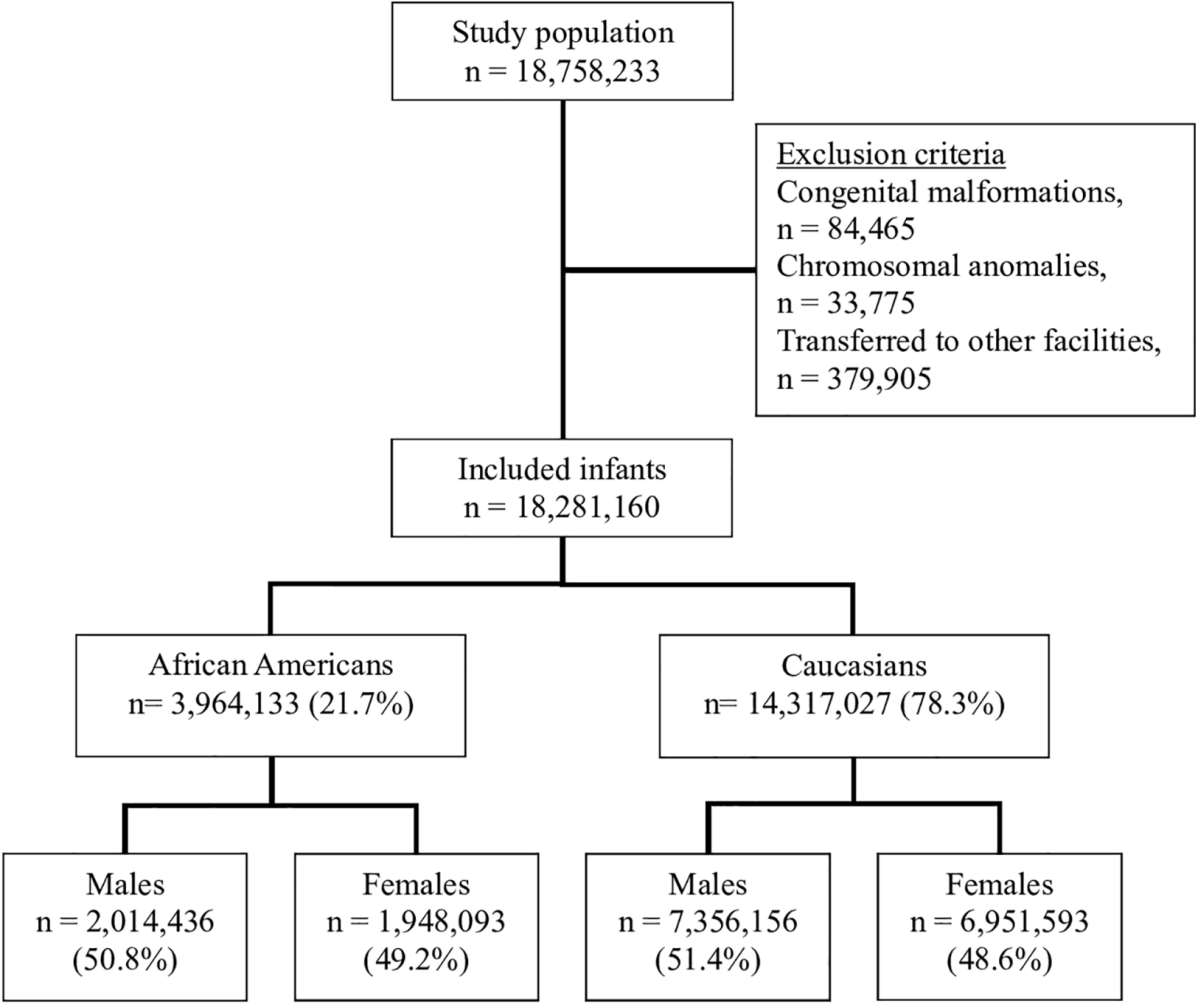
Study population.

Weighted sample in the dataset for the years 2011–2018 included 18,758,233 infants; 78.3% of them were C and 21.7% were AA. Female infants represented 51.3%, full-term infants were 91.8% and overall mortality was 0.3%. The flow chart of the study population is presented with inclusion and exclusion criteria in Figure 1. The demographic and clinical characteristics of the study population are shown in Table 1.

**Table 1:** Demographic characteristics and outcomes of study population.

	African Americans *n* = 3,964,133	Caucasianss *n* = 14,317,027	OR (CIs), *p*-value
Female sex^a^	49.2	48.6	1.02 (1.02–1.03), <0.01
Singleton^a^	96.4	96.6	0.94 (0.93–0.95), <0.01
Birth weight <2,500 g^a^	9.1	4.9	1.93 (1.92–1.94), <0.01
Gestational age			
<24 weeks	12,823 (0.32)	13,148 (0.09)	3.53 (3.45–3. 26), <0.01
24 weeks	5,815 (0.15)	6,833 (0.05)	3.08 (2.97–3. 91), <0.01
25–26 weeks	13,748 (0.35)	17,611 (0.12)	2.82 (2.76–2.88), <0.01
27–28 weeks	18,268 (0.46)	27,904 (0.19)	2.37 (2.33–2.42), <0.01
29–30 weeks	24,827 (0.63)	44,724 (0.31)	2.01 (1.98–2.05), <0.01
31–32 weeks	42,712 (1.08)	92,534 (0.65)	1.67 (1.66–1.69), <0.01
33–34 weeks	97,030 (2.45)	246,440 (1.72)	1.43 (1.42–1.44), <0.01
35–36 weeks	212,157 (5.35)	616,319 (4.30)	1.26 (1.25–1.26), <0.01
≥37 weeks	3,536,752 (89.2)	13,251,514 (92.6)	0.67 (0.66–0.67), <0.01
Length of stay (day)^b^	4.5 (2)	3.5 (2)	<0.01
Mortality^a^	0.56	0.23	2.47 (2.43–2.52), <0.01

^a^ Data are presented as total number (percentage) except where indicated with ^a^where it is percentage only.

^b^ Data are presented as mean and (median).

The overall mortality rate in C was 0.23% and in AA infants was 0.56% (*p* = 0.01). Mortality was highest at earlier GA < 24 weeks (84.1%) and lowest at term GA (0.09%). Mortality in AA was significantly higher in infants ≥36 weeks compared to C infants. However, racial/ethnic disparity in mortality was not consistent in preterm infants. In male infants, AA infant had less mortality at GA < 24 weeks through 28 weeks and at 35–36 weeks; mortality did not differ between AA and C at 29–34 weeks. The overall aOR for mortality in AA in all male preterm infants ≤36 weeks was 1.44 (1.39–1.49), <0.01, Table 2. In female infants, AA had less mortality than C at GA 24–26 weeks and 31–34 weeks; it did not differ at GA < 24 weeks, 27–30 weeks, and 35–36 weeks. Weeks. The overall aOR for mortality in AA in all preterm female infants ≤36 weeks was 1.38 (1.33–1.44), <0.01, Table 2.

**Table 2:** Mortality in African American and Caucasian infants by gestational age.

Panel (A): Mortality in African American versus Caucasian male infants				
	African Americans *n* = 2,014,436	Caucasians *n* = 7,356,156	OR (CIs), *p*-value	Adjusted OR, *p*-value^a^
Overall	12,467(0.63)	18,243 (0.28)	1.89 (1.72.-2.16), <0.01	1.40 (1.36-1.45), <0.01
<24 weeks	5937 (85.6)	6162 (86.5)	0.92 (0.84-1.01), 0.09	0.85 (0.73-0.97), =0.02
24 weeks	1133 (38.3)	1666 (44.0)	0.79 (0.72-0.87), <0.01	0.80 (0.70-0.91), <0.01
25-26 weeks	1074 (15.5)	1631 (17.5)	0.87 (0.80-0.95), <0.01	0.76 (0.69-0.83), <0.01
27-28 weeks	460 (5.11)	919 (6.31)	0.80 (0.71-0.90), <0.01	0.69 (0.61-0.78), <0.01
29-30 weeks	272 (2.15)	559 (2.31)	0.93 (0.80-1.03), 0.34	0.90 (0.77-1.06), =0.21
31-32 weeks	225 (1.05)	440 (0.89)	1.19 (1.01-1.40), 0.04	1.19 (1.00-1.43), =0.05
33-34 weeks	215 (0.44)	547 (0.41)	1.06 (0.91-1.25), 0.44	0.94 (0.79-1.11), =0.45
35-36 weeks	156 (0.15)	487 (0.15)	0.98 (0.82-1.17), 0.82	0.75 (0.62-0.91), <0.01
>36 weeks	2959 (0.16)	5832 (0.09)	1.92 (1.83-2.00), <0.01	1.52 (1.45-1.60), <0.01
Panel (B): Mortality in African American versus Caucasian female infants				
	African Americans *n* = 1,948,093	Caucasians *n* = 6,951,593	OR (CIs), *p*-value	Adjusted OR^a^
Overall	9765 (0.54)	14,232(0.24)	1.74 (1.69.-2.05), <0.01	1.47 (1.41-1.53), <0.01
<24 weeks	4634 (79.8)	4962 (83.3)	0.79 (0.72-0.87), <0.01	0.88 (0.77-1.01), =0.07
24 weeks	819 (28.7)	1097 (36.3)	0.71 (0.63-0.79), <0.01	0.61 (0.53-0.70), <0.01
25-26 weeks	917 (13.5)	1278 (15.4)	0.86 (0.78-0.94), <0.01	0.77 (0.70-0.86), <0.01
27-28 weeks	401 (4.36)	627 (4.72)	0.92 (0.81-1.05), 0.20	0.87 (0.76-1.01), =0.06
29-30 weeks	242 (1.99)	384 (1.88)	1.06 (0.90-1.25), 0.46	0.94 (0.79-1.13), =0.51
31-32 weeks	139 (0.65)	416 (0.97)	0.67 (0.55-0.81), <0.01	0.60 (0.48-0.73), <0.01
33-34 weeks	124 (0.26)	440 (0.39)	0.68 (0.55-0.81), <0.01	0.67 (0.54-0.83), <0.01
35-36 weeks	172 (0.16)	379 (0.13)	1.26 (1.05-1.50), 0.01	1.05 (0.87-1.28), =0.58
>36 weeks	2317 (0.13)	4649 (0.07)	1.85 (1.76-1.95), <0.01	1.49 (1.41-1.58), <0.01
Viability check: *n*, % and OR after excluding mortality within first 48 hours in <24 week infants				
	African Americans	Caucasians	OR (CIs), *p*-value	Adjusted OR, *p*-value^a^
<24 weeks Males	766 (43.3)	849 (46.9)	0.86 (0.75-0.98), 0.03	0.87 (0.72-1.04), =0.13
<24 weeks Females	638 (35.2)	639 (39.2)	0.84 (0.73-0.97), 0.02	0.86 (0.73-1.03), =0.10

Data are presented in number (%).

^a^ Regression analysis was used adjusting for 3 maternal factors (hypertension, diabetes, and chorioamnionitis) and 12 neonatal factors (sex, birthweight category, occurrence of hypoxic ischemic insults, intraventricular hemorrhage, periventricular leukomalacia, respiratory distress syndrome, persistent pulmonary hypertension, pulmonary hemorrhage, bronchopulmonary dysplasia, sepsis, necrotizing enterocolitis, and primary type of insurance).

**Figure 2:**
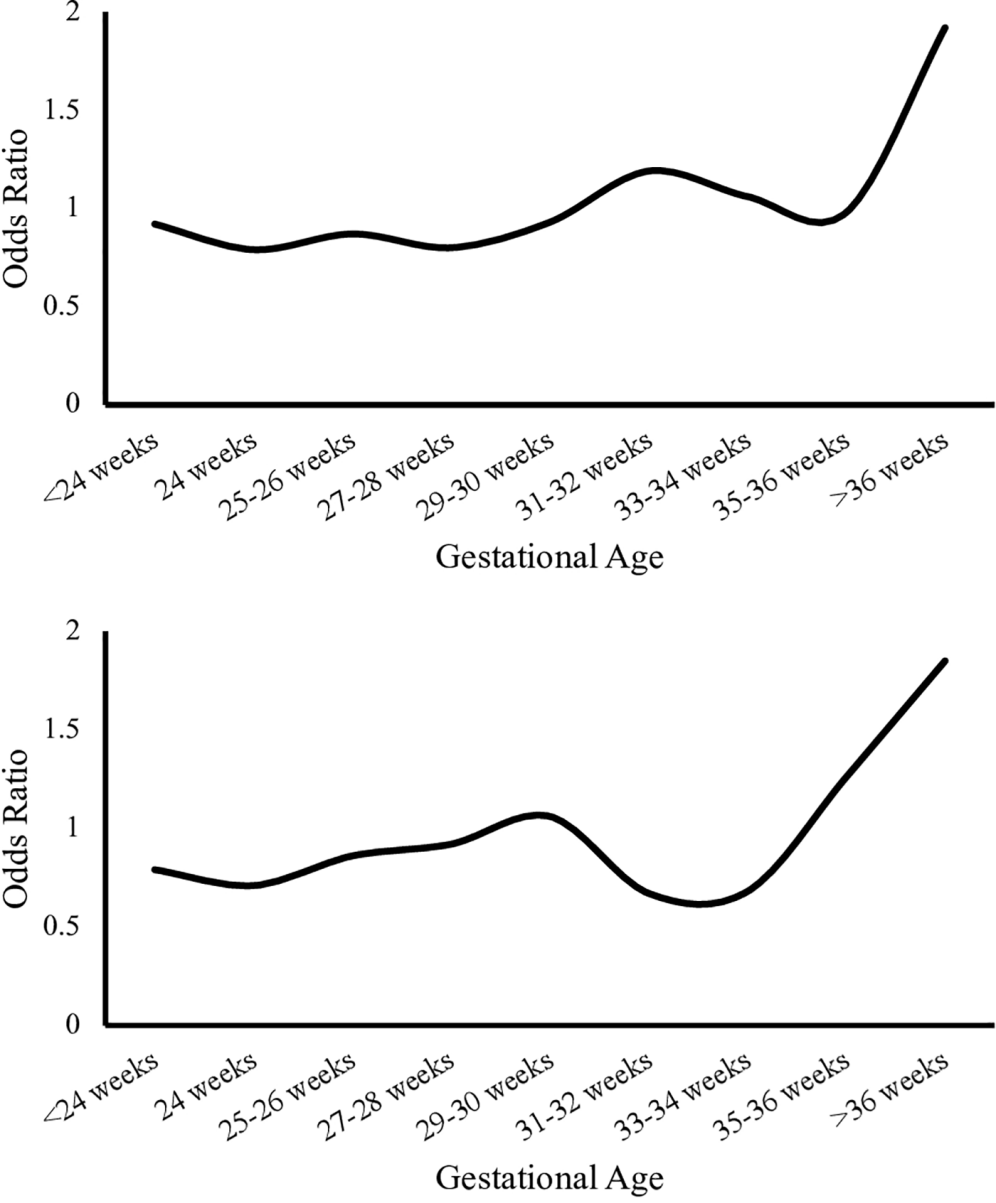
Odds ratio for mortality in Caucasian infants compared to African American infants by gestational age. Upper panel represents odds ratio in Caucasian compared to African American in males. The lower panel represents odds ratio in Caucasian compared to African American in females.

Females had lower mortality compared to males [0.27% vs. 0.33%, OR: 0.82 (0.81–0.84), *p* < 0.01] in the overall population, [0.20% vs. 0.25%, OR: 0.83 (0.81–0.84), *p* < 0.01] in C infants and [0.50% vs. 0.62%, OR 0.81 (0.79–0.83), *p* < 0.01] among AA infants. There was higher mortality among AA compared to C male infants [0.62% vs. 0.25%, OR: 2.50 (2.44–2.56), *p* < 0.01] and that was also true among female population [0.50% vs. 0.20%, OR: 2.46 (2.39–2.52), *p* < 0.01], Table 2. Figure 2 presents the odds ratio for mortality in AA compared to C infants in each sex category.

Analyses were repeated after excluding neonates born at the edge of viability with GA < 24 weeks who died in the first 48 h. Repeated analysis showed consistent ratios with findings above, where mortality among AA compared to C male infants born <24 weeks was 43.3% vs. 46.9% while the OR remained 0.86 (0.75–0.98), *p* = 0.03. Similarly, mortality among AA compared to C female infants born <24 weeks was 35.2 vs. 39.2 with OR: 0.84 (0.73–0.97), *p* = 0.02.

Regression analyses were performed to account for neonatal and maternal confounding variables with adjusted OR displayed in Table 2. Mortality in AA was significantly higher in term infants born at GA ≥ 37 weeks when compared to C infants. Mortality among preterm AA was either less than or similar to mortality among C preterm infants born at GA 24–36 weeks. Mortality in neonates born at the edge of viability with GA < 24 weeks did not differ between AA and C in the male and female populations (Table 2).

## 4 Discussion

This study demonstrated the GA timeline of racial/ethnic disparities in neonatal mortality. In the overall population, C population, and AA population, males had higher mortality compared to females. Racial/Ethnic disparity was tested with each sex separately. Compared to C, there was increased mortality in full term AA male and female infants born at GA > 36 weeks. Disparity existed in the opposite direction with less mortality in AA compared to C in preterm male and female infants born at GA 24–26 weeks. Disparity did not exist in periviable male and female infants born at GA < 24 weeks.

We showed that, in infants born ≤36 weeks of gestation, AA population did not have consistent disparity in mortality when compared to C population born at the same gestational age. For example, in male infants, AA infant had less mortality at GA < 24 weeks through 28 weeks and at 35–36 weeks; mortality did not differ between AA and C at 29–34 weeks. In female infants, AA had less mortality than C at GA 24–26 weeks and 31–34 weeks; it did not differ at GA < 24 weeks, 27–30 weeks, and 35–36 weeks. Weeks. However, the overall mortality was higher in AA male preterm infants ≤36 weeks [aOR = 1.44 (1.39–1.49), <0.01] and the overall aOR for mortality in AA in all preterm female infants ≤36 weeks was 1.38 (1.33–1.44), <0.01.

Previous maternal demographic data has shown that C women had a higher fetal mortality rate compared to AA women for gestational ages <32 weeks, and a shift occurred at ≥32 weeks with AA women having higher fetal mortality rates (7).

This study demonstrated that, compared to C population, there was significantly higher mortality in AA infants born >36 weeks in females and males. There was a previous report that described increased infant mortality in AA population in a single year [2007] for gestational age ≥34 weeks compared to other racial/ethnic groups (8). The results in the current study indicate that disparities in infant mortality start at >36 weeks, providing insight into the timeline of fetal programming and epigenetic mechanisms. Based on the timing of appearance of racial disparities, it is biologically plausible that epigenetics could have a role in this process. Epigenetics have been previously explored to explain variation in infant mortality regarding fetal development. The fetus responds to the changes in intrauterine environment and makes physiological adaptations in response to nutritional stimuli or fetal glucocorticoid exposure through fetal programming (9). Epigenetic mechanisms, particularly DNA methylation, are modified in response to exposures like psychosocial stress. DNA methylation has been demonstrated to have an association with preterm birth and gestational age (10). It has been shown that AA experience higher levels of adverse social and environmental exposures, and DNA methylation could play a role in observed ethnic disparities in health (10). Previous studies have shown that DNA methylation advances during late fetal development (11). This correlates with the results of this study where disparities begin at 33 weeks gestational age, providing a point of interest into when epigenetic factors influence health outcomes of infants.

Psychosocial stressors affecting mother can affect the intrauterine environment and the growing fetus. The Barker Hypothesis proposed that suboptimal in-utero environment generates fetal stress that presents with low birth weight and causes increased susceptibility to metabolic syndrome later in life (12). AA undergo more psychosocial stresses, measured by recent life events, chronic stress, traumatic events, and discrimination stress, than their C counterparts (13). Geronimus's concept of weathering suggests that AA women experience early health deterioration due to the cumulative effects of repeated experiences with social, economic, or political barriers (14). AA women, specifically have higher allostatic loads, defined as the cumulative wear and tear on the body due to repeated adaption to stressors, compared to AA males and C women across all ages (15). A greater exposure to lasting stressors enforces exhaustive coping mechanisms and deterioration of the biological systems in this population (12). Taking into consideration the demonstrated relationship between stressors and AA women, there is a great need to explore how this relates to observed ethnic disparities in infant mortality.

It is imperative to consider the social aspects of health for birthing mothers and infants and how it is related to ethnic disparities. There is an inverse association between discrimination and health, and there is a need to measure health-related aspects of racism to assess its stressful dimensions and the mechanisms that connect discrimination to health (16). Persistent ethnic disparities in infant mortality raise concern for the root social causes of these outcomes. Clark et al., argue that inequities experienced by marginalized and racialized communities will continue unless efforts are made to address social and structural determinants of health and root causes, including racism, that contribute to unequal outcomes (17). Antenatal variables within the social determinants of health (SDH) affect access to medical care and influence an individual's capacity for healthy living, which may affect neonatal hospital outcomes. Further research examining the pathway between SDH and ethnic disparities in infant mortality is recommended. Racial disparate neonatal care could be a plausible explanation for increased mortality in infants >36 weeks of gestation. Several studies have shown such effect; the current study yet provides more substance to this ongoing discussion (16, 18).

The strengths of this study include that it is a national database study. This study is the first to compare ethnic disparities within infant mortality by GA. One limitation of this study is that a national de-identified dataset does not follow up the subjects as out-patient. Some deaths that occurred after hospital discharge, but within the 28-day period that defines neonatal mortality, may not have been accounted for. Although the data set included information about 3 maternal variables, it did not include full maternal background information, some of which may have been relevant in influencing/affecting neonatal outcomes. Another limitation is that the “Other” category for the race variables includes infants that had an unknown race which could have skewed results if their race was known.

## 5 Conclusion

AA population has significantly higher mortality in female neonates born >34 weeks and males born >36 weeks of gestation. The mortality in preterm infant population demonstrated inconsistent results. Male infants had marginally lower mortality compared to females in the overall population. This study provides essential data that is needed to devise preventative prenatal care given the origin of ethnic disparities which become evident in neonates born around 35 weeks gestational age. Further studies are recommended to address fetal mortality combined with maternal conditions. In addition, future research is needed to identify the timeline of epigenetic mechanisms during fetal development and determine if there is a link to neonatal mortality.

## Data Availability

The original contributions presented in the study are included in the article/Supplementary Material, further inquiries can be directed to the corresponding author.
